# Primary Spinal Epidural Diffuse Large B-cell Lymphoma: Case Report and Literature Review

**DOI:** 10.7759/cureus.28934

**Published:** 2022-09-08

**Authors:** Prarthna V Bhardwaj, Annie Abraham, Sunitha Alluri

**Affiliations:** 1 Hematology Oncology, University of Massachusetts Medical School Baystate, Springfield, USA; 2 Pathology and Laboratory Medicine, University of Massachusetts Medical School Baystate, Springfield, USA

**Keywords:** primary spinal epidural lymphoma, dlbcl, psel, non-hodgkin lymphoma, primary spinal lymphoma

## Abstract

Primary spinal epidural lymphoma (PSEL) comprises a group of tumors present only in the spinal epidural space with a histopathological picture of lymphoma and negative diagnostic workup for lymphoma at other sites. We present the case of an older male adult with primary spinal diffuse large B-cell lymphoma (DLBCL) presenting with spinal cord compression who was treated with surgery followed by high dose methotrexate in combination with RCHOP (rituximab, cyclophosphamide, prednisone, vincristine, and doxorubicin). This case report and review of literature on DLBCL limited to the spine provide a novel chemotherapy regimen and a comprehensive perspective on the optimal management of these patients.

## Introduction

Primary spinal epidural lymphoma (PSEL) accounts for 0.9%-6.5% of all extranodal NHLs [1,2]. Spinal epidural diffuse large B-cell lymphoma (DLBCL) is the most common form, accounting for less than 2% of all DLBCLs [3].

The pathophysiology of spinal epidural lymphomas is poorly understood. Lymphomas are thought to arise from the paravertebral ganglions or the epidural lymphoid tissues [3]. The encroachment into the epidural space occurs through the intervertebral foramina without the erosion of the surrounding bone tissue. There is a male preponderance of PSEL with the thoracic spine being the most involved site [1]. This is attributed to the rich venous plexus in the area, greater length of the thoracic spine compared with cervical and lumbar, and more tolerance for bulky disease in the thorax and abdomen.

The most common presenting symptoms include back pain, lower extremity weakness, and bladder dysfunction, with a subacute onset ranging from days to weeks. Imaging alone does not allow reliable discrimination from the other epidural neoplasms, including metastasis, meningioma, and multiple myeloma or inflammatory and infectious causes.

There are various reports of this entity; however, there is no consensus on the optimal management of these patients [1,4-11]. We present a patient with primary spinal epidural diffuse large B-cell lymphoma presenting with cord compression managed with a combination of surgery and a novel chemotherapy regimen resulting in improved functional outcomes.

## Case presentation

A 73-year-old male presented to the hospital with progressive leg weakness for four weeks. He reported worsening mid-back pain with progressive sensory and motor weakness in his bilateral lower extremities. He was independent of his activities of daily living until four weeks before presentation and eventually became wheelchair-bound over a month. He did not sense the urge to urinate and had urinary incontinence. He denied any stool abnormalities. He had a diminished sensation below the level of his umbilicus.

He had a medical history significant for chronic lumbar pain, not requiring any analgesic or hypertriglyceridemia but was otherwise healthy. He lived with his family without any recent travel outside of his area of residence. He used to smoke cigarettes in the past but quit 25 years ago. He did not report any alcohol or recreational drug use. He reported no family history of cancer.

On presentation, his vitals were within normal limits. Physical examination was notable for intact motor and sensory exam in the upper extremities but was paraplegic in his lower extremities along with diminished sensations below the level of the umbilicus. He had no palpable lymphadenopathy. Cardiac and pulmonary examinations were regular. Labs obtained at the time of admission were grossly unremarkable (Table 1).

**Table 1:** Laboratory parameters WBC: white blood cell, AST: aspartate transaminase, ALT: alanine transaminase, PSA: prostate specific antigen, Ig: Immunoglobulin, LDH: lactate dehydrogenase, HIV: human immunodeficiency virus

Lab parameters	Values (units)
Hemoglobin	13.3 g/dL
WBC count	9000/mm^3^
Platelet count	184,000/mm^3^
Creatinine	0.9 mg/dL
AST	16 u/L
ALT	14 u/L
Alkaline Phosphatase	67 u/L
Total Bilirubin	0.4 mg/dL
PSA	1.2 ng/mL
IgG	1,024 mg/dL
IgA	345 mg/dL
IgM	51mg/dL
Serum immunofixation	No evidence of monoclonal protein
LDH	289 u/L
HIV testing	Negative

**Figure 1:**
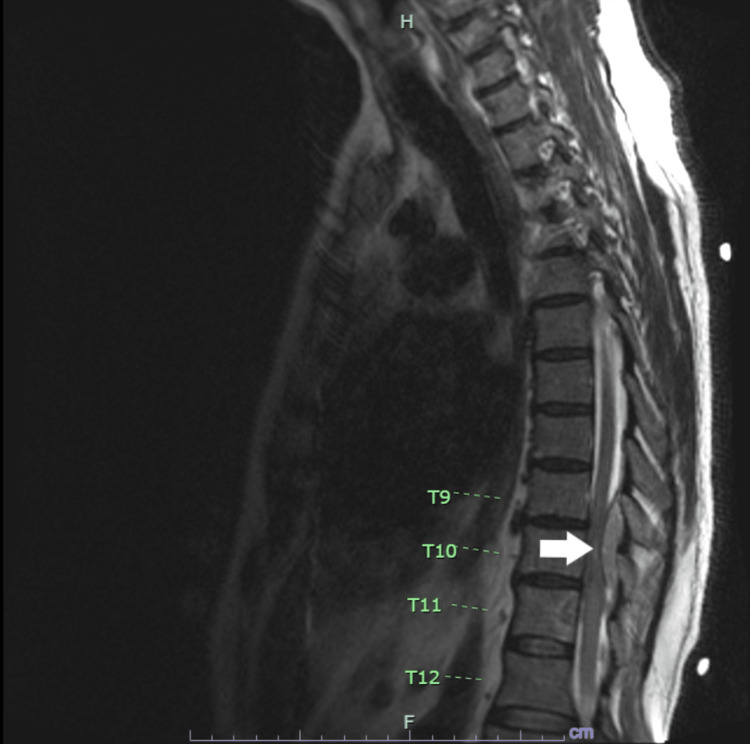
Sagittal T2 mildly hyperintense and enhancing soft tissue within the epidural space at mid T9 extending inferiorly through T12 (see white arrow)

He had an MRI of the lumbar spine done, with and without contrast, that showed mild degenerative changes without any high-grade stenosis or nerve root compression. He then had an MRI of the thoracic spine with and without contrast which showed multilevel enhancing epidural soft tissues at T9-T12 with severe central canal narrowing and spinal cord compression at lower T9 to the upper half T11. Soft tissue extension into multiple foramina was noted with differential considerations including but not restricted to metastatic disease, lymphoma, and granulomatous disease (Figure 1). No pathological compression fracture was reported in the thoracic or lumbar spine and there was no cauda equina compression.

**Figure 2:**
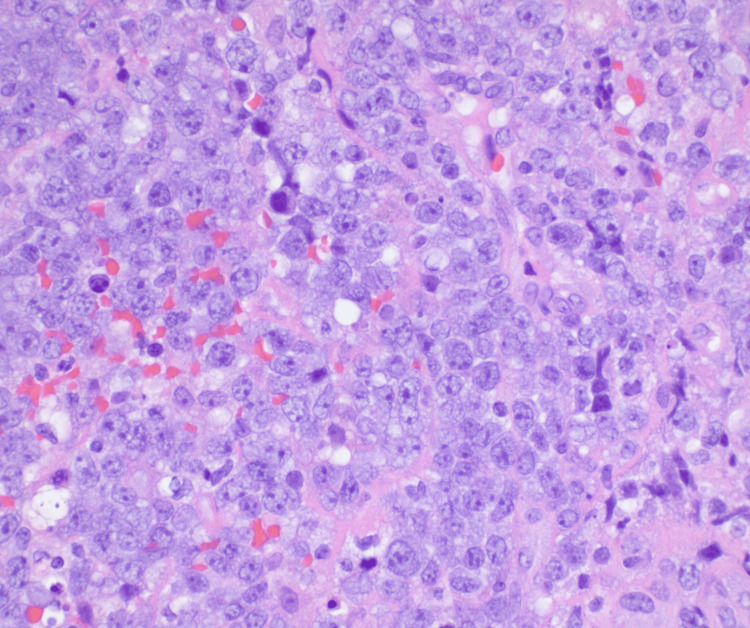
60x magnification; pleomorphic tumor cells with amphophilic cytoplasm, large nuclei, and one or multiple variably prominent nucleoli.

**Figure 3:**
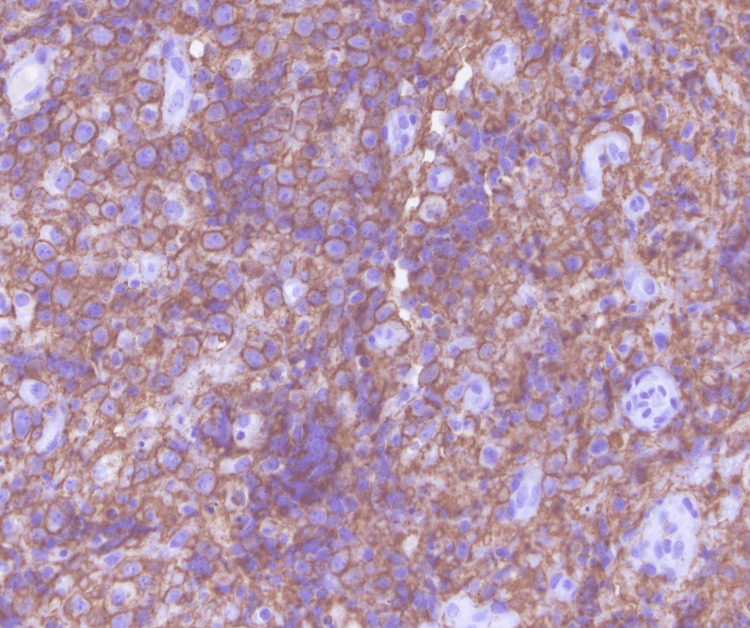
60x magnification; diffuse membranous immunoreactivity with CD20 in lymphoma cells.

**Figure 4:**
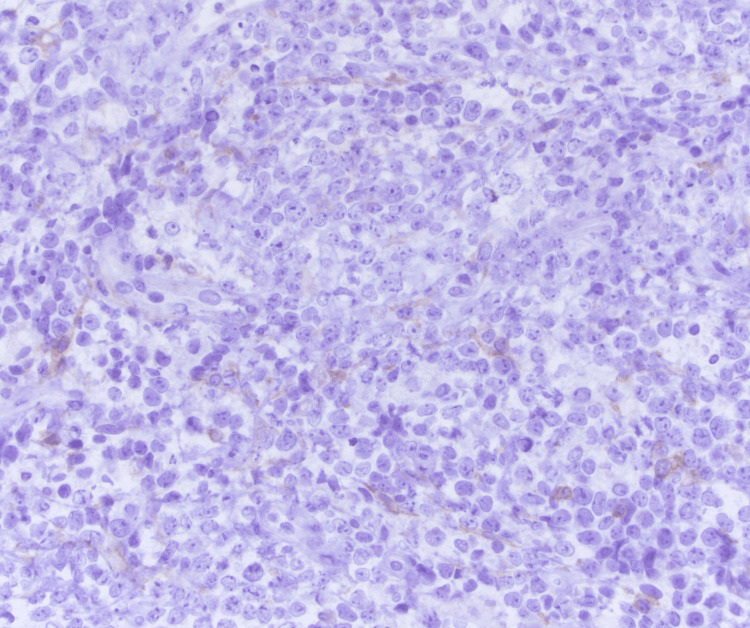
60x magnification; minimal immunoreactivity with CD10 (interpreted as negative)

**Figure 5:**
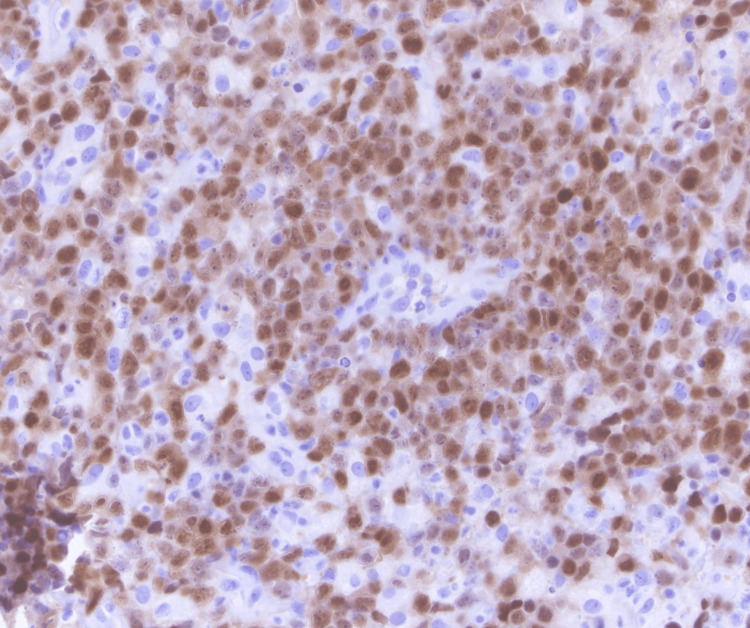
60x magnification; diffuse nuclear immunoreactivity with MUM1 in lymphoma cells (greater than 30% staining considered positive)

**Figure 6:**
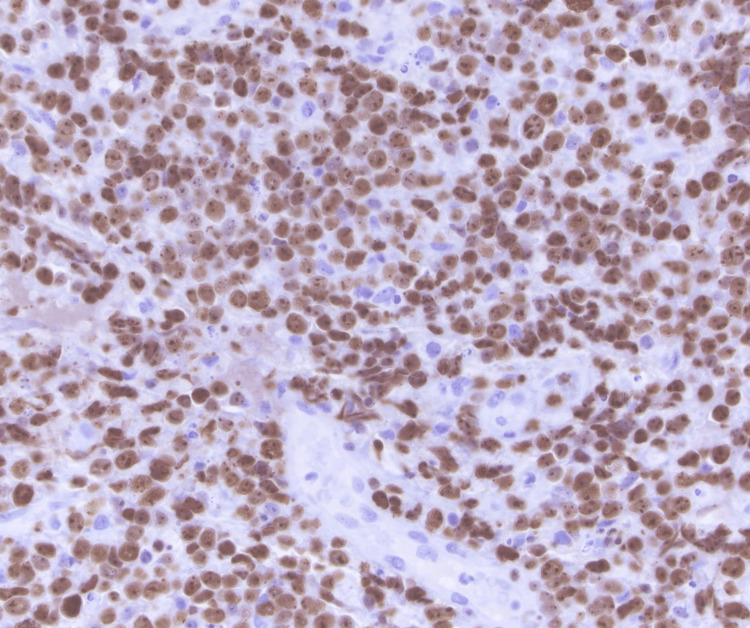
Diffuse nuclear immunoreactivity for Ki-67 in the majority of lymphoma cells (proliferation index of around 90%).

He underwent a CT of the chest abdomen pelvis with contrast that showed mild splenomegaly with otherwise no evidence of metastatic disease within the abdomen or pelvis. He underwent decompression surgery with T9-T10 laminectomy for resection of epidural spinal cord tumor with frozen sections revealing malignant neoplasm. He was noted to have a residual epidural tumor extending into the neural foramina from T9-T12 and extension into T11. There was also persistent tumor encircling and compressing the spinal cord at T11 with myelopathic cord changes at T10 and T11. The histology from his surgical specimen was positive for CD20, PAX-5, BCL-6, MUM1, and BCL-2 with a negative keratin cocktail, S100, GFAP, and CD3. Ki-67 was around 90% (Figures 2-6). This was consistent with a diagnosis of diffuse large B-cell lymphoma nongerminal center type with no c-MYC, BCL-2, or BCL-6 rearrangements. He underwent a bone marrow biopsy that was negative for lymphoma.

Given his neurological deficits, he could not have an inpatient PET CT scan due to the urgent need to initiate chemotherapy. Given the lack of other disease sites, he was presumed to have primary spinal epidural lymphoma.

He received chemotherapy with an MR-CHOP regimen for six cycles every 21 days, which included rituximab 375mg/m2 IV on Day 1, high dose methotrexate 3.5mg/m2 IV on Day 1, followed by leucovorin rescue, doxorubicin 50mg/m2 IV on Day 4, vincristine 2mg IV on Day 4, cyclophosphamide 750mg/m2 IV on Day 4 and prednisone 100mg daily by mouth from Day 1 to 5. Given his age, he was also given pegfilgrastim subcutaneously on Day 6 of every cycle.

After completing three cycles of treatment, he underwent an interim PET scan, which showed no evidence of lymphoma consistent with Deauville’s score of one on his scan. He continued to have borderline splenomegaly of 14cm, which remained unchanged without any focal lesions. After completing six cycles of treatment, the PET scan continued to show an ongoing response to treatment with a Deauville score of two. He was referred to radiation oncology for localized radiation given his initial presentation; however, he did not complete it due to a lack of timely follow-up.

His clinical course was complicated by neutropenic fever related to Escherichia coli bacteremia associated with a urinary tract infection from a Foley catheter. He also developed another neutropenic fever episode after his second treatment cycle about herpes simplex stomatitis. He tolerated the rest of his treatment without any other side effects or complications.

At his six-monthly follow-up, he can transfer himself off his wheelchair and stand up from a sitting position with support. His lower extremity strength continues to improve with rehabilitation. A repeat PET scan at the end of six months did not show any ongoing evidence of lymphoma. 

## Discussion

Primary spinal DLBCL remains a rare entity. Age >50 years, male sex, thoracic location, patients with more aggressive histological types, and the lack of combined modality treatment are thought to have a worse prognosis [1,9]. Local control of 88% and overall survival of 69% were observed with combined modality treatment of surgery and radiation based on one of the surveys of this rare cancer [1]. A study from China evaluating 130 patients with PSEL of different histology, including B-cell lymphomas, T-cell lymphomas, and Burkitt’s lymphoma, reported a three-year overall survival of 81.1% [9].

No specific management has been established for this entity due to its low incidence. Chang et al. demonstrated that primary surgical decompression improved neurological outcomes in spinal cord compression due to DLBCL [4]. However, other studies recommended non-surgical management of cord compression related to spinal lymphoma, given higher postoperative mortality [5,6]. As no patients are worse functionally after surgery, we believe surgical intervention is warranted at least to establish a definite pathological diagnosis. The combination of chemotherapy and radiation is more effective than either alone. Harris et al. recommended an innovative algorithm in which primary neurosurgical intervention is pursued in the presence of severe neurological deficit or if tissue diagnosis is needed. In other cases, primary chemoradiation can be attempted, and surgical intervention can be performed in case of clinical deterioration [8]. Our patient presented with debilitating neurological symptoms, and hence, underwent surgery to improve his neurological outcome. There was a plan for consolidative radiation following the completion of chemotherapy; however, he missed the therapeutic window due to a delay in follow-up. Based on a review of published case reports, Table 2 lists the characteristics and modality of treatment used in patients with PSEL, specifically the DLBCL type.

**Table 2:** Published case reports about primary spinal epidural DLBCL DLBCL: diffuse large B-cell lymphoma; R-CHOP: rituximab, cyclophosphamide, doxorubicin, vincristine, and prednisone; CHOP: cyclophosphamide, doxorubicin, vincristine, and prednisone; MR-CHOP: high dose methotrexate, cyclophosphamide, doxorubicin, vincristine, and prednisone; N/A: not applicable.

Author, Year	Patient Characteristics	Location of tumor	Modality of treatment	Choice of chemotherapy
Pokhrel et al., 2020 [10]	24yo M	T10-L2	Surgery, chemotherapy	RCHOP
Pinheiro et al., 2020 [11]	65yo M	T1-T4	Surgery, chemotherapy, radiation	MR-CHOP (dose not specified)
Liu et al., 2018 [12]	43yo M	T5	Surgery	N/A
Cordoba-Mosqueda et al., 2017 [13]	45yo M	T3	Surgery, chemotherapy, radiation	Not specified
Moussaly et al., 2015 [14]	65yo F	T4-T9, T11	Surgery, chemotherapy, radiation	RCHOP followed by intrathecal methotrexate
Cho et al., 2015 [15]	29yo F	L5-S2	Surgery, chemotherapy	RCHOP followed by maintenance chemotherapy with Rituximab for an unspecified duration
Jagtap et al., 2013 [16]	32yo F	T3-T4	Surgery, chemotherapy	Not specified
Mally et al., 2011 [17]	24yo M	L5-S2	Surgery, chemotherapy, radiation	CHOP
Kapoor et al., 2006 [18]	28yo F	L3	Radiation, chemotherapy	CHOP

While there is an increased risk of CNS relapse for patients with extradural disease in the setting of disseminated lymphoma, it is not clear if patients with localized disease carry the same risk. We propose the addition of high-dose methotrexate to patients with this entity due to the high penetrance of this medication in the central nervous system, which could possibly limit CNS relapse. This was successfully demonstrated by Masaki et al. in a pilot study of seven patients utilizing a similar regimen for patients with primary central nervous system lymphoma with a 100% overall response rate [19]. Our patient is the first reported case of using 3.5g/m2 of high dose methotrexate as part of the chemotherapy regimen despite his older age which he tolerated without other significant concerns.

## Conclusions

In conclusion, considering PSEL in the differential diagnosis of space-occupying lesions of the vertebral canal presenting with spinal cord compression is pertinent. This remains a rare diagnosis without a standard approach to treatment. However, we recommend a combined modality approach including surgery, chemotherapy, and radiation for optimal outcomes based on our extensive literature review. High dose methotrexate dosed at 3.5gm/m2 can be added to conventional CHOP in the treatment of DLBCL of the spine. 
